# Transcriptomic profiles of the ruminal wall in Italian Mediterranean dairy buffaloes fed green forage

**DOI:** 10.1186/s12864-023-09215-6

**Published:** 2023-03-20

**Authors:** Angela Salzano, Salvatore Fioriniello, Nunzia D’Onofrio, Maria Luisa Balestrieri, Riccardo Aiese Cigliano, Gianluca Neglia, Floriana Della Ragione, Giuseppe Campanile

**Affiliations:** 1 Department of Veterinary Medicine and Animal Production, Federico II University, Naples, Italy; 2 Institute of Genetics and Biophysics ‘A. Buzzati-Traverso’, CNR, Naples, Italy; 3 Department of Precision Medicine, University of Campania Luigi Vanvitelli, Naples, Italy; 4 Sequentia Biotech, SL, Carrer de València, 08009 Barcelona, Spain; 5 IRCCS Istituto Neurologico Mediterraneo Neuromed, Pozzilli, Isernia Italy

**Keywords:** Transcriptome, Green feed, Rumen, Buffalo, RNA-seq

## Abstract

**Background:**

Green feed diet in ruminants exerts a beneficial effect on rumen metabolism and enhances the content of milk nutraceutical quality. At present, a comprehensive analysis focused on the identification of genes, and therefore, biological processes modulated by the green feed in buffalo rumen has never been reported. We performed RNA-sequencing in the rumen of buffaloes fed a total mixed ration (TMR) + the inclusion of 30% of ryegrass green feed (treated) or TMR (control), and identified differentially expressed genes (DEGs) using EdgeR and NOISeq tools.

**Results:**

We found 155 DEGs using EdgeR (*p*-values < 0.05) and 61 DEGs using NOISeq (prob ≥0.8), 30 of which are shared. The rt-qPCR validation suggested a higher reliability of EdgeR results as compared with NOISeq data, in our biological context.

Gene Ontology analysis of DEGs identified using EdgeR revealed that green feed modulates biological processes relevant for the rumen physiology and, then, health and well-being of buffaloes, such as lipid metabolism, response to the oxidative stress, immune response, and muscle structure and function. Accordingly, we found: *(i)* up-regulation of *HSD17B13*, *LOC102410803* (or *PSAT1*) and *HYKK*, and down-regulation of *CDO1*, *SELENBP1* and *PEMT*, encoding factors involved in energy, lipid and amino acid metabolism; *(ii)* enhanced expression of *SIM2* and *TRIM14*, whose products are implicated in the immune response and defense against infections, and reduced expression of *LOC112585166* (or *SAAL1*), *ROR2*, *SMOC2,* and *S100A11*, encoding pro-inflammatory factors; *(iii)* up-regulation of *NUDT18*, *DNAJA4* and *HSF4*, whose products counteract stressful conditions, and down-regulation of *LOC102396388* (or *UGT1A9*) and *LOC102413340* (or *MRP4/ABCC4*), encoding detoxifying factors; *(iv)* increased expression of *KCNK10, CACNG4,* and *ATP2B4*, encoding proteins modulating Ca^2+^ homeostasis, and reduced expression of the cytoskeleton*-*related *MYH11* and *DES.*

**Conclusion:**

Although statistically unpowered, this study suggests that green feed modulates the expression of genes involved in biological processes relevant for rumen functionality and physiology, and thus, for welfare and quality production in Italian Mediterranean dairy buffaloes.

These findings, that need to be further confirmed through the validation of additional DEGs, allow to speculate a role of green feed in the production of nutraceutical molecules, whose levels might be enhanced also in milk.

**Supplementary Information:**

The online version contains supplementary material available at 10.1186/s12864-023-09215-6.

## Introduction

In mammals, a homeostatic orchestration of several metabolic processes in the different tissues is required for the maintenance of lactation. In particular, the gastrointestinal tract is the central site of feed digestion, nutrient uptake, and has a pivotal role in the endocrinal control of ruminant metabolism [1]. Exploring the biology of the different tissues and organs of gastrointestinal system, including their gene expression profiles and metabolic pathways, may significantly increase the knowledge of the ruminant biology and lead to discovering new potential candidate genes for future increases in animal production, in terms of both quantity and quality. Several studies have demonstrated the relevance of RNA sequencing (RNA-seq) and other omics approaches for the identification of metabolic processes potentially involved in lactating dairy cows [2–4]. It has been reported that milk composition could be influenced by many factors such as breed, diseases, environment, parity, days in milk and many others [5]. Among these factors, the diet, whether or not mediated by the rumen metabolism, has a pivotal role since it can deeply modify the organoleptic and nutraceutical quality of both milk and dairy products. Several studies have demonstrated that pasture-based diets can positively influence milk nutrient profile improving the concentration of several beneficial compounds such as Omega-3 polyunsaturated fatty acids, vaccenic and linolenic acid, and conjugated linoleic acid (CLA), as well as reducing the levels of Omega-6 fatty acids and palmitic acid [6–10].

Rumen metabolic processes have a significant impact on the composition of milk nutrients. The products of the rumen fermentation, such as short-chain fatty acids (SCFAs) produced in the forestomaches, indeed, are largely absorbed across the epithelium of the rumen and of the omasum [11] and, together with other nutraceutical molecules, are extracted into the blood, metabolized by the liver and finally transported to the mammary gland [3]. Of note, SCFAs meet approximately 80% of the energy needs for these animals, and therefore, they play important roles in the maintenance of energy homeostasis of ruminants [12].

Improving the nutraceutical properties of milk is an attempt to meet consumers demand of healthy, “natural”, and sustainable dairy products. Consumers have started to ask for “natural” foods, and this could be made possible modifying milk composition directly at the farm stage through dietary intervention, without using mechanical modifications such as processing, fat separation, ultrafiltration, etc. [8, 13, 14]. When it is not possible to use pastures, the utilization of green feed could represent a low-cost opportunity. It has already been seen that the inclusion of green forage in the diet enhances the content of some components with nutraceutical properties [15], such as vaccenic and rumenic acids [16], and betaines and carnitines [17]. In nature, feed regimens have the potential to induce changes in the transcriptional program of the cells, often through the modulation of the epigenome, and this could have a significant impact on many biological processes, such as metabolism, health and development [18]. Angus beef cattle that received a grass-based diet showed changes in the rumen expression of gene networks associated with immune mechanisms, cellular development, and the biosynthesis of key molecules [19]. In buffalo species, few molecular studies have been performed so far, taking advantage of RNA-seq and other omics approaches [20, 21], and no information is available on the impact of different feed regimens on the transcriptomic landscape in these species. In a previous study [17], it has been shown that the administration of green forage in Italian Mediterranean dairy buffaloes enhanced the antioxidant and antineoplastic activity of buffalo milk. Based on this evidence, it is conceivable that the provision of green feed to Italian Mediterranean dairy buffaloes would also induce, in the rumen, changes in the expression of genes whose products participate to networks linked to the metabolism of important biomolecules.

The aim of the present study was to identify in rumen the impact of green feed diet on molecular mechanisms relevant for rumen functionality and physiology, through the analysis of ruminal transcriptomic profiles of buffaloes that received a standard total mixed ration (TMR) or a TMR + ryegrass green feed (30% of diet). The obtained results might highlight the beneficial effect of this diet on welfare-related molecular processes, and contribute to elucidate the role of green feed in the production of nutraceutical molecules, whose levels might be enhanced also in milk.

## Results

### Transcriptomic analysis in the ruminal wall of dairy buffaloes

RNA-seq yielded an average of 47,967,818 high-quality 150 bp reads (23,983,909 paired reads) per sample. Approximately 93% of the reads were mapped to the *Bubalus bubalis* reference genome sequence (version UOA_WB_1 from NCBI) and almost 84% of the reads were uniquely mapped (Additional file 1: Table S1). A total of 18,386 and 18,380 genes were expressed (Trimmed Mean of M values, TMM > 1) in ruminal wall of buffaloes that received TMR + green feed (treated group) and TMR (control group), respectively (Additional file 2: Table S2).

### Green feed re-modulates the expression of several genes in rumen of dairy buffaloes

The identification of differentially expressed genes (DEGs) between treated and control groups (six animals for each condition) has been carried out using two different approaches: EdgeR and NOISeq software packages.

**Fig. 1:**
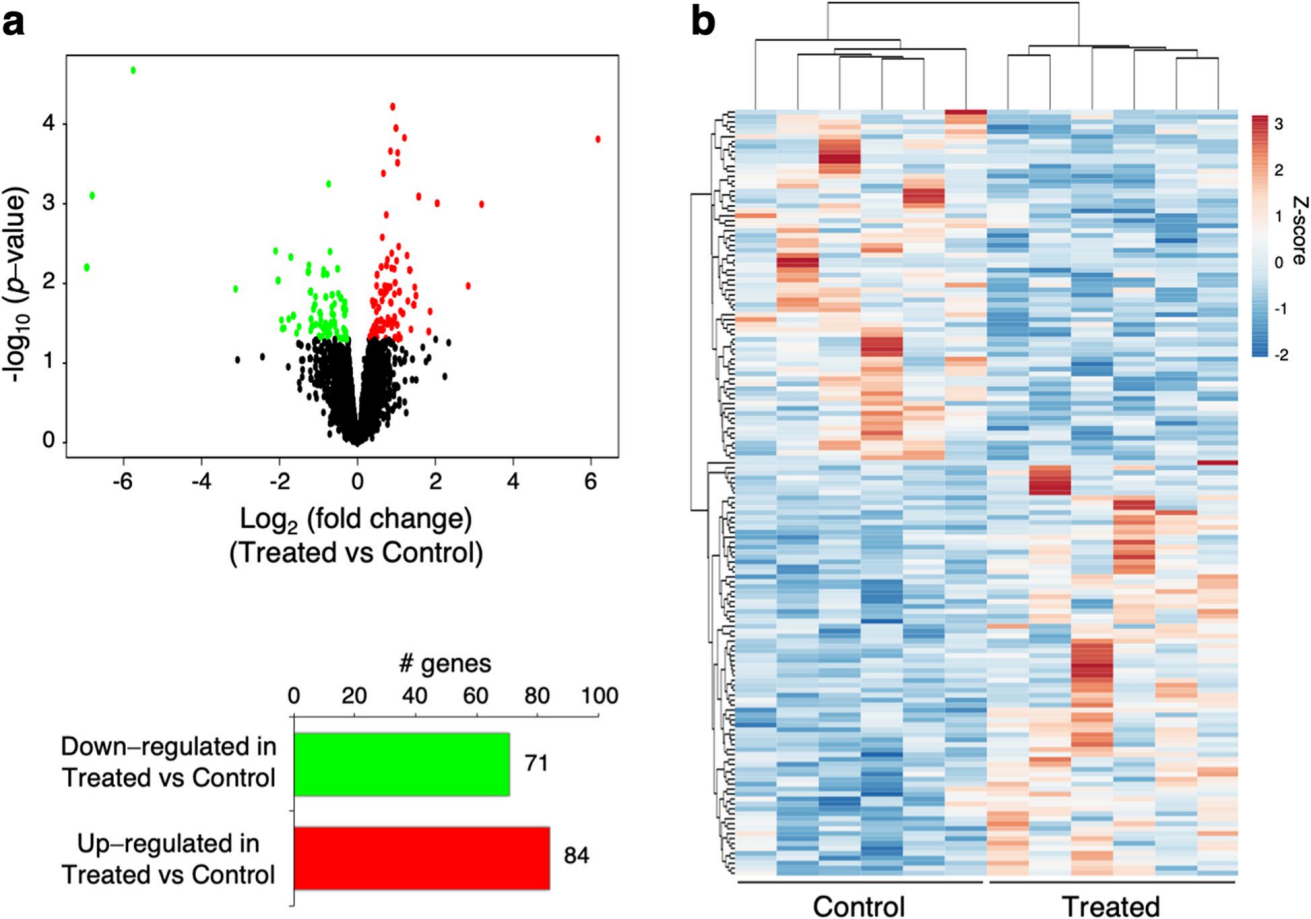
Effect of green feed on transcriptional profile in rumen of dairy buffaloes (six animals for each condition), identified using EdgeR (nominal *p*-values < 0.05). **a** Top: Volcano plot that illustrates differentially expressed genes (DEGs) in rumen of dairy buffaloes fed a TMR + green feed (Treated) versus animals fed the TMR diet (Control). The significance of the differential expression (−log_10_ (*p*-value*)*, y-axis) is plotted versus fold change expression level (x-axis). Red and green dots depict the genes that are significantly up- and down-regulated in treated versus control animals; black dots are genes whose expression levels did not reach the statistical significance (*p*-value *< 0.05)* between the two experimental groups. Bottom: Bar graph that shows the number of up- and down-regulated genes in treated vs control animals. **b** Heatmap representation of significant DEGs (155) across all the samples. Blue-red gradient shows low to high expression levels for each gene. Dendrogram represents a hierarchical clustering based on Pearson correlation values

Initial analysis of DEGs performed using EdgeR with FDR-adjusted *p*-values < 0.05 as significance threshold failed to yield significant DEGs. Considering that the two diets were isonitrogenous and isoenergetic, and varied only for the inclusion of green forage, we expect small variations of gene expression following the change of feed regimen, that could be masked by the inter-individual variability of the animals belonging to the same group. In the light of this hypothesis, we decided to identify DEGs using nominal *p*-values < 0.05 as significance threshold. This analysis identified 155 DEGs (Fig. 1; Additional file 3: Table S3). The volcano plot depicts the statistical significance of the differential expression between the two groups and the difference in gene expression levels (Fig. 1a, top). Among DEGs, 71 genes showed lower expression and 84 displayed higher expression in the rumen of treated buffaloes (Fig. 1a, bottom). In Fig. 1b is shown the heatmap that illustrates, through a hierarchical clustering analysis, the significant DEGs across all the samples.

Among the genes found to be deregulated in rumen of treated versus control animals, using EdgeR software package, the top 20 up-regulated (Table 1) and down-regulated (Table 2) genes were selected in terms of the magnitude of differential expression, log_2_ (fold change), to identify the processes being affected by the different diet composition.

**Table 1:** Top up-regulated genes in rumen of treated vs control dairy buffaloes identified using EdgeR tool (nominal *p*-values < 0.05) tool. (FC: fold change)

Gene name	Protein name	log_2_ (FC)	-log_10_ (*p*-value)	*p*-value
*MKRN3*	probable E3 ubiquitin-protein ligase makorin-3	2.84	1.97	1.07E-02
*LOC102407663*	interferon alpha-inducible protein 27-like protein 2 isoform X1	2.05	3.01	9.85E-04
*SIM2*	single-minded homolog 2 isoform X1	1.83	1.40	4.01E-02
*LOC102408976*	interferon alpha-inducible protein 27, mitochondrial	1.57	3.09	8.11E-04
*C2H6orf15*	uncharacterized protein C6orf15 homolog isoform X2	1.5	1.85	1.42E-02
*HSD17B13*	17-beta-hydroxysteroid dehydrogenase 13	1.47	1.95	1.12E-02
*CSTA*	cystatin-A	1.37	1.42	3.77E-02
*LOC102411389*	pantetheinase isoform X3	1.34	2.17	6.78E-03
*SLC46A2*	thymic stromal cotransporter homolog	1.27	2.35	4.46E-03
*LOC102395439*	four and a half LIM domains protein 1, partial	1.21	3.83	1.48E-04
*PRR19*	proline-rich protein 19 isoform X2	1.17	1.62	2.38E-02
*CACNG4*	voltage-dependent calcium channel gamma-4 subunit	1.11	1.65	2.23E-02
*CCDC85A*	coiled-coil domain-containing protein 85A isoform X5	1.08	1.90	1.27E-02
*IGFBP6*	insulin-like growth factor-binding protein 6	1.06	2.46	3.44E-03
*FCER2*	low affinity immunoglobulin epsilon Fc receptor isoform X3	1.04	1.31	4.94E-02
*LOC102397197*	tubulin alpha-1D chain	1.03	3.64	2.29E-04
*DUOXA1*	dual oxidase maturation factor 1 isoform X2	1.03	3.51	3.06E-04
*RTL5*	retrotransposon Gag-like protein 5 isoform X1	1.02	1.48	3.31E-02
*TPPP3*	tubulin polymerization-promoting protein family member 3	0.99	3.95	1.12E-04
*IFI44L*	interferon-induced protein 44-like isoform X1	0.97	2.01	9.79E-03

**Table 2:** Top down-regulated genes in rumen of treated vs control dairy buffaloes identified using EdgeR tool (nominal *p*-values < 0.05). (FC: fold change)

Gene name	Protein name	log_2_ (FC)	-log_10_ (*p*-value)	*p*-value
*LOC112585166*	serum amyloid A protein-like	−5.75	4.68	2.10E-05
*LRP2*	LOW QUALITY PROTEIN: low-density lipoprotein receptor-elated protein 2	−3.13	1.93	1.17E-02
*NYAP2*	neuronal tyrosine-phosphorylated phosphoinositide-3-kinase adapter 2 isoform X1	−2.04	2.04	9.19E-03
*LOC102389855*	cytochrome c oxidase subunit 7C, mitochondrial	−1.88	1.44	3.63E-02
*MUC15*	mucin-15	−1.76	1.55	2.80E-02
*LOC102399021*	cytochrome P450 1A1	−1.71	2.33	4.67E-03
*LEF1*	lymphoid enhancer-binding factor 1 isoform X5	−1.65	1.60	2.54E-02
*TTPA*	alpha-tocopherol transfer protein	−1.56	1.38	4.20E-02
*LOC102399215*	nuclear distribution protein nudE homolog 1-like	−1.5	1.46	3.48E-02
*ZNF618*	zinc finger protein 618 isoform X21	−1.27	2.15	7.15E-03
*ROR2*	tyrosine-protein kinase transmembrane receptor ROR2	−1.25	2.23	5.89E-03
*CADPS*	calcium-dependent secretion activator 1 isoform X17	−1.2	1.90	1.27E-02
*SMOC2*	LOW QUALITY PROTEIN: SPARC-related modular calcium-binding protein 2	−1.2	1.41	3.91E-02
*LOC102400888*	olfactory receptor 51E1 isoform X2	−1.19	1.55	2.80E-02
*IZUMO1*	izumo sperm-egg fusion protein 1 isoform X5	−1.16	1.47	3.41E-02
*HSPA12A*	heat shock 70 kDa protein 12A isoform X2	−1.13	1.68	2.09E-02
*PODN*	podocan	−1.09	1.73	1.85E-02
*LOC112578513*	multidrug resistance-associated protein 4-like	−1.08	1.83	1.47E-02
*SYNPO2*	synaptopodin-2 isoform X2	−0.99	1.34	4.61E-02
*SMARCA1*	probable global transcription activator SNF2L1 isoform X3	−0.96	1.63	2.34E-02

Included in the top up-regulated genes, we found *LOC102407663 (or interferon alpha-inducible protein 27*-*like protein 2*, *IFI27L2*), *single*-*minded homolog 2 protein* (*SIM2*) and *interferon*-*induced protein 44*-*like (IFI44L)* genes, which are associated with immune response; the lipid metabolism-related *17*-*beta*-*hydroxysteroid dehydrogenase 13* (*HSD17B13*) and *LOC102411389* (or *Vanin 1*, *VNN1*) genes; *LOC102395439* (or *four and a half LIM domains protein 1*, *FHL1*), *tubulin polymerization*-*promoting protein family member 3 (TPPP3),* and *LOC102397197 (or tubulin alpha-1D chain, TUBA1D) *genes, whose products are involved in cytoskeletal organization; the low-affinity IgE receptor *Fc Epsilon Receptor II* (*FCER2*) gene; the oxidative stress- and immune system-linked *dual oxidase maturation factor 1 isoform X2* (*DUOXA1*) gene; *insulin*-*like growth factor-binding protein 6* (*IGFBP6*) gene.

Among the top down-regulated genes, we identified the *LOC112585166* (or *serum amyloid A protein*-*like*, *SAAL1*), *tyrosine*-*protein kinase transmembrane receptor* (*ROR2*), and *SPARC*-*related modular calcium*-*binding protein 2* (*SMOC2*) genes, encoding pro-inflammatory factors; the *LDL receptor related protein 2* (*LRP2*) and *LOC102399021* (or *cytochrome P450 1A1*, *CYP1A1*) genes, which are related to lipid metabolism; the energy metabolism-linked *LOC102389855* (or *cytochrome C oxidase subunit 7C, Cox7C*) gene; the *LOC102399215* (or *nuclear distribution protein nudE homolog 1*-*like*, *NDEL1*) gene, involved in cytoskeletal organization; *LOC112578513* (or *multidrug resistance*-*associated protein 4*-*like* (*ABCC4*) gene, involved in the response to oxidative stress; the extracellular matrix*-*associated *synaptopodin-2 (SYNPO2)* gene; *LOC102400888* (or *olfactory receptor 51E1, OR51E1*) gene; the transporter of α-tocopherol (vitamin E) *alpha tocopherol transfer protein* (*TTPA*) gene; the membrane-bound glycosylated *Mucin*-*15 (MUC15*) gene.

**Fig. 2:**
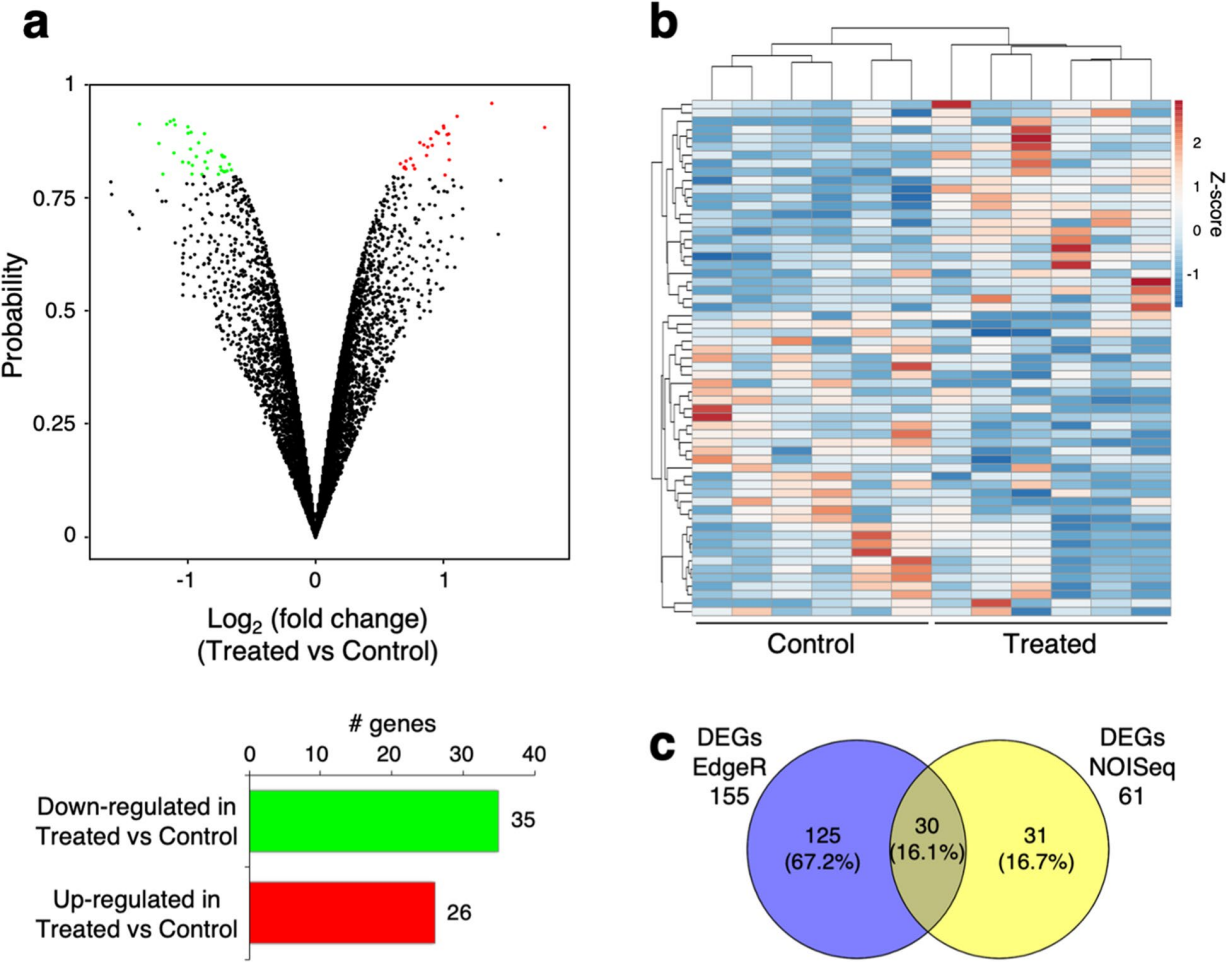
Effect of green feed on transcriptional profile in rumen of dairy buffaloes (six animals for each condition), identified using NOISeq software (prob *≥*0.8). **a** Top: Volcano plot that illustrates differentially expressed genes (DEGs) in rumen of dairy buffaloes fed a TMR + green feed (Treated) versus animals fed the TMR diet (Control). The significance of the differential expression (probability, y-axis) is plotted versus fold change expression level (x-axis). Red and green dots depict the genes that are significantly up- and down-regulated in treated versus control animals; black dots are genes whose expression levels did not reach the statistical significance (prob ≥0.8) between the two experimental groups. Bottom: Bar graph that shows the number of up- and down-regulated genes in treated vs control animals. **b** Heatmap representation of significant DEGs (61) across all the samples. Blue-red gradient shows low to high expression levels for each gene. Dendrogram represents a hierarchical clustering based on Pearson correlation values. **c** Venn diagram that shows the overlap between DEGs identified with EdgeR (nominal *p*-values < 0.05) and NOISeq (prob *≥*0.8) tools

A parallel analysis, performed using the NOISeq software package with a posterior probability (prob) ≥ 0.8 as significance threshold, identified 61 DEGs, 35 of which showed lower expression and 26 displayed higher expression in the rumen of treated buffaloes (Fig. 2a). Among DEGs, 11 of these have a prob. ≥0.9 and only 1 DEG shows a prob. ≥0.95 (Additional file 3: Table S3). The statistical significance of the differential expression between the two groups and the difference in gene expression levels are illustrated with volcano plot (Fig. 2a). The heatmap reported in Fig. 2b illustrates the significant DEGs with a prob. ≥0.8 across all the samples, through a hierarchical clustering analysis.

The comparison of DEGs identified with EdgeR and NOISeq software packages highlighted 30 genes that showed a coherent differential expression in treated vs control groups using the two different approaches (Fig. 2c), 3 of which with a prob. ≥0.9 (Additional file 3: Table S3).

In Tables 3 and 4 are reported the top 10 up-regulated and down-regulated genes in terms of the magnitude of differential expression, log_2_ (fold change), identified in rumen of treated versus control animals using NOISeq software package.

**Table 3:** Top up-regulated genes in rumen of treated vs control dairy buffaloes identified using NOISeq tool (prob ≥0.8). (FC: fold change)

Gene name	Protein name	log_2_ (FC)	probability
*LOC112583793*	WAP four-disulfide core domain protein 18-like	1.37	0.96
*LOC102397197*	tubulin alpha-1D chain	1.10	0.93
*IGFBP6*	insulin-like growth factor-binding protein 6	1.04	0.89
*LOC102411389*	pantetheinase isoform X3	1.03	0.89
*DUOXA1*	dual oxidase maturation factor 1 isoform X2	1.01	0.80
*TCN1*	transcobalamin-1	1.00	0.90
*TPPP3*	tubulin polymerization-promoting protein family member 3	1.00	0.91
*BMP6*	bone morphogenetic protein 6 isoform X1	0.91	0.87
*PNLDC1*	poly(A)-specific ribonuclease PNLDC1 isoform X3	0.90	0.88
*LAMA1*	LOW QUALITY PROTEIN: laminin subunit alpha-1	0.86	0.84

**Table 4:** Top down-regulated genes in rumen of treated vs control dairy buffaloes identified using NOISeq tool (prob ≥0.8). (FC: fold change)

Gene name	Protein name	log_2_ (FC)	probability
*ACSS3*	acyl-CoA synthetase short-chain family member 3, mitochondrial isoform X1	−1.37	0.91
*ROR2*	tyrosine-protein kinase transmembrane receptor ROR2	−1.22	0.87
*SMOC2*	LOW QUALITY PROTEIN: SPARC-related modular calcium-binding protein 2	−1.19	0.80
*FRAS1*	extracellular matrix protein FRAS1 isoform X3	−1.16	0.91
*WTIP*	Wilms tumor protein 1-interacting protein isoform X2	−1.10	0.92
*ITGA8*	integrin alpha-8 isoform X2	−1.10	0.85
*LOC112580184*	glutathione S-transferase A1	−1.09	0.91
*LOC102400339*	collagen alpha-1(I) chain-like	−0.99	0.91
*TSKS*	testis-specific serine kinase substrate isoform X9	−0.99	0.83
*TENM3*	LOW QUALITY PROTEIN: teneurin-3	−0.97	0.86

Among the top up-regulated genes, we identified *LOC102397197 (or TUBA1D), IGFBP6, LOC102411389 (or VNN1), DUOXA1, TPPP3, bone morphogenetic protein 6* (*BMP6), poly(A)-specific ribonuclease PNLDC1* (*PNLDC1*), and *laminin subunit alpha-1* (*LAMA1*) genes, which are also included in the list of up-regulated genes identified using EdgeR software (see above and Additional file 3: Table S3). In addition, among the top up-regulated genes identified by NOISeq analysis, we found also *LOC112583793* (or *WAP four-disulfide core domain protein 18-like*, *WFDC18)* gene, encoding a proteinase inhibitor, and *transcobalamin-1* (*TCN1*) gene, encoding a vitamin B12-binding protein.

Included in the top down-regulated genes, we found *ROR2, SMOC2, LOC102400339* (or *collagen alpha-1(I) chain-like, COL1A1*) genes, and the *teneurin-3* (*TENM3*) gene, encoding a transmembrane protein, all down-regulated also with EdgeR analysis (see above and Additional file 3: Table S3). Moreover, the list of the top down-regulated genes identified with NOISeq analysis includes also *acyl-CoA synthetase short-chain family member 3* (*ACSS3*) gene, whose product has a role in energy and lipid metabolism, *fraser extracellular matrix complex subunit 1* (*FRAS1*) gene, *integrin alpha-8* (*ITGA8*) gene, *Wilms tumor protein 1-interacting protein* (*WTIP*) gene, encoding a factor involved in the response to hypoxia, and *LOC112580184* (or *glutathione S-transferase A1, GSTA1*) gene, whose product has a role in the response to the oxidative stress.

The lists of all DEGs in treated group in comparison with the control one, identified using EdgeR and NOISeq software packages, and the DEGs identified with both approaches (common DEGs) are reported in Additional file 3: Table S3.

To validate the differential expression observed by RNA-seq analysis, the expression of randomly selected DEGs identified with EdgeR and NOISeq software packages was analyzed in rumen of treated and control buffaloes by reverse-transcription qPCR (rt-qPCR), using the same samples analyzed by RNA-seq. We analyzed the expression of 12 DEGs identified with EdgeR tool and 8 DEGs identified with NOISeq tool, 4 of which resulted commonly deregulated in RNA-seq experiment analyzed with both approaches.

**Fig. 3:**
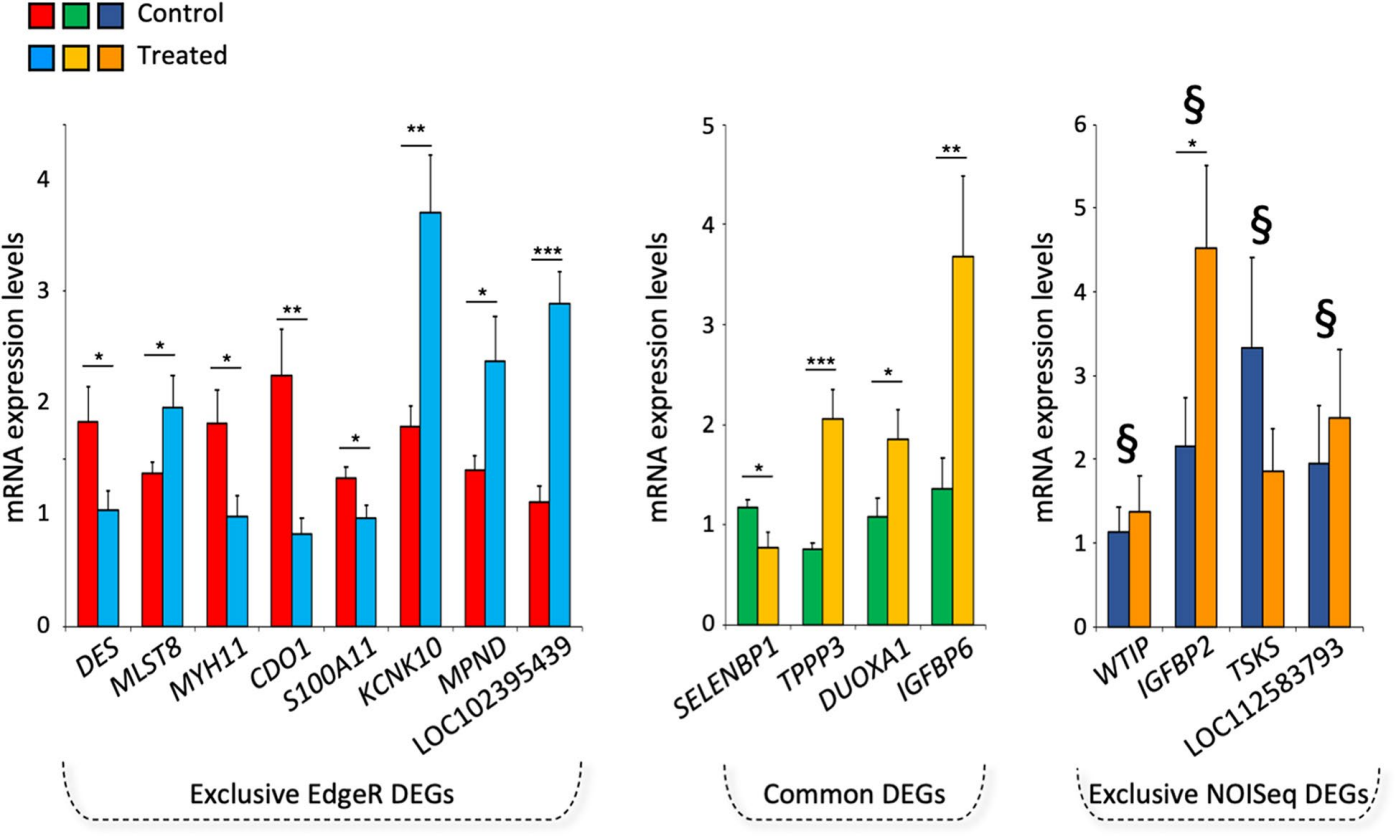
Validation of the differential expression observed by RNA-seq. The expression of randomly selected DEGs [12 DEGs identified with EdgeR tool and 8 DEGs identified with NOISeq tool, 4 of which are deregulated with both approaches (common DEGs)] was analyzed by rt-qPCR in ruminal wall of dairy buffaloes fed a TMR + green feed (Treated) in comparison with animals fed the TMR diet (Control). Data were normalized to β-actin and are reported as mean fold increases ± standard error of the mean (SEM) relative to transcript levels of control group samples. Six control and six treated animals were used, and two independent qPCR experiments were performed. * *p* < 0.05; * *p* < 001; *** *p* < 0.001 (one-tailed Student’s t-test). §: rt-qPCR data conflicting with RNA-seq results

For all 12 genes identified with EdgeR tool (nominal *p*-values < 0.05), the rt-qPCR assays showed an expression pattern concordant with RNA-seq results (Fig. 3). On the other hand, among randomly selected genes found to be deregulated with NOISeq analysis, differential expression of genes identified with both methods [common DEGs: *selenium binding protein 1* (*SELENBP1*), *DUOXA1*, and *IGFBP6* with 0.8 < prob. < 0.9; *TPPP3* with 0.9 < prob. < 0.95] has been validated by rt-qPCR assay, whereas DEGs identified only with NOISeq analysis [*LOC112583793* (or *WFDC18)* with prob. > 0.95; WTIP with 0.9 < prob. < 0.95; *insulin-like growth factor-binding protein 2* (IGFBP2) and *testis-specific serine kinase substrate* (*TSKS*) with 0.8 < prob. < 0.9] showed an expression pattern conflicting with RNA-seq data. These findings suggest a higher reliability of EdgeR data analysis with respect to the NOISeq approach in our experimental conditions, and prompted us to perform subsequent analyses using DEGs selected with EdgeR approach.

### Gene ontology analysis of differentially expressed genes

Gene Ontology Enrichment Analysis (GOEA) was undertaken on the 155 DEGs between treated versus controls buffaloes, identified using EdgeR tool. Overall, DEGs were functionally associated with 431 significantly enriched gene ontology (GO) terms (FDR-adjusted *p*-value < 0.05), 195 of which were enriched in up-regulated genes and 236 were enriched in down-regulated genes (Additional file 4: Table S4).

**Fig. 4:**
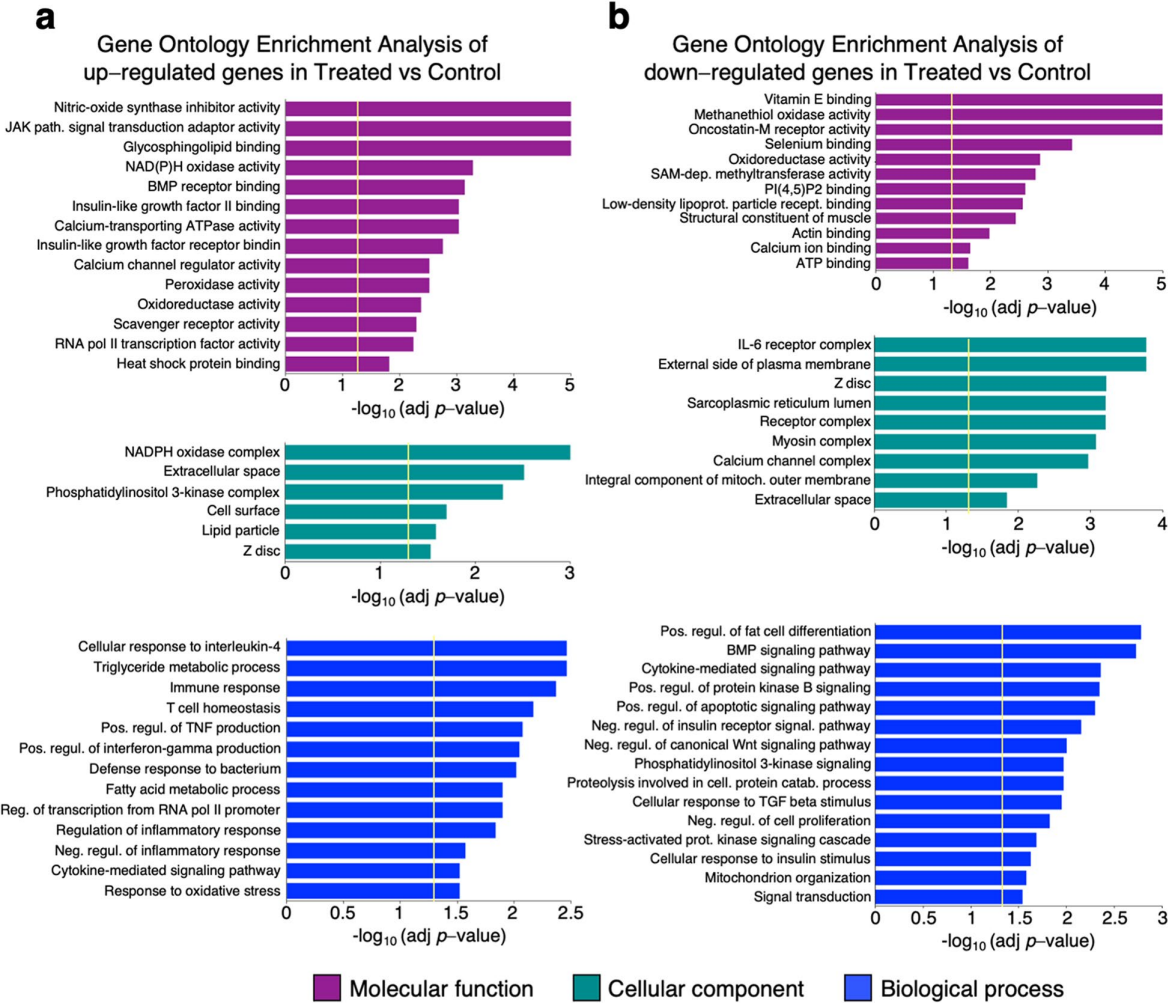
Gene ontology enrichment analysis of differentially expressed genes. Selected GO-terms, enriched in genes up-regulated (**a**) and down-regulated (**b**) in rumen of buffaloes fed a TMR + green feed (Treated) in comparison with those fed the TMR diet (Control), are shown. GO terms were classified as molecular function (purple bars), cellular component (green bars) and biological process (blue bars). Bar graphs indicate the statistical significance of the enrichment, as -log_10_ (adj *p*-value). Vertical yellow bars indicate the cut-off level for significance (*p* < 0.05, adjusted by Benjamini-Hochberg correction)

The 35% (68) of GO terms enriched in up-regulated genes were classified as molecular function, 17% (33) as cellular component, and 48% (94) as biological process. The 38% (89) of GO terms enriched in down-regulated genes were categorized as molecular function, 22% (51) as cellular component, and 40% (96) as biological process. Selected GO terms enriched in up- and down-regulated genes are displayed in Fig. 4a and b. Among GO terms enriched in up-regulated genes, the category of molecular functions included those related to nitric oxide production, JAK-STAT pathway, NAD(P)H oxidase activity, peroxidase and oxidoreductase activity, calcium channel activity and heat shock proteins. In the category of cellular components we found GO terms related to extracellular space and Z Disc - a component of the striated muscle with a key role for the muscle contraction -, while the category of biological processes included GO terms linked to immune system (cellular response to interleukin-4, immune response, T cell homeostasis, positive regulation of interferon-gamma production, defense response to bacterium and cytokine-mediated signaling pathway and negative regulation of inflammatory response), to lipid metabolism (triglyceride metabolic process and fatty acid metabolic process) and to the response to oxidative stress. Down-regulated genes were enriched in molecular function GO terms related to methanethiol oxidase activity, to selenium homeostasis (selenium binding), to oxidoreductase activity, to S-adenosyl methionine (SAM)-dependent methyltransferase activity, and to structural constituent of muscle. In the category of cellular components, we found an enrichment in down-regulated genes in GO terms related to the external side of plasma membrane, Z disc and calcium channel complex. The category of biological processes included GO terms associated with positive regulation of fat cell differentiation, with BMP signaling pathway, with cytokine-mediated signaling pathway, with positive regulation of apoptotic signaling pathway, and with negative regulation of canonical *Wnt* signaling pathway.

In summary, the Gene Ontology analysis indicated that green feed modulated biological processes related to health and well-being in buffaloes, such as the immune response, lipid metabolism, the response to the oxidative stress and muscle structure and function.

### Gene-by-gene analysis of differential expression

Further analysis of individual genes found to be differentially expressed in rumen of treated dairy buffaloes in comparison with control animals, using EdgeR with a nominal *p*-values < 0.05, allowed us to highlight transcriptional changes in genes involved in biological processes relevant for rumen functionality and physiology, and thus, for animal welfare and quality production.

#### Genes linked to energy, lipid and amino acid metabolism

**Fig. 5:**
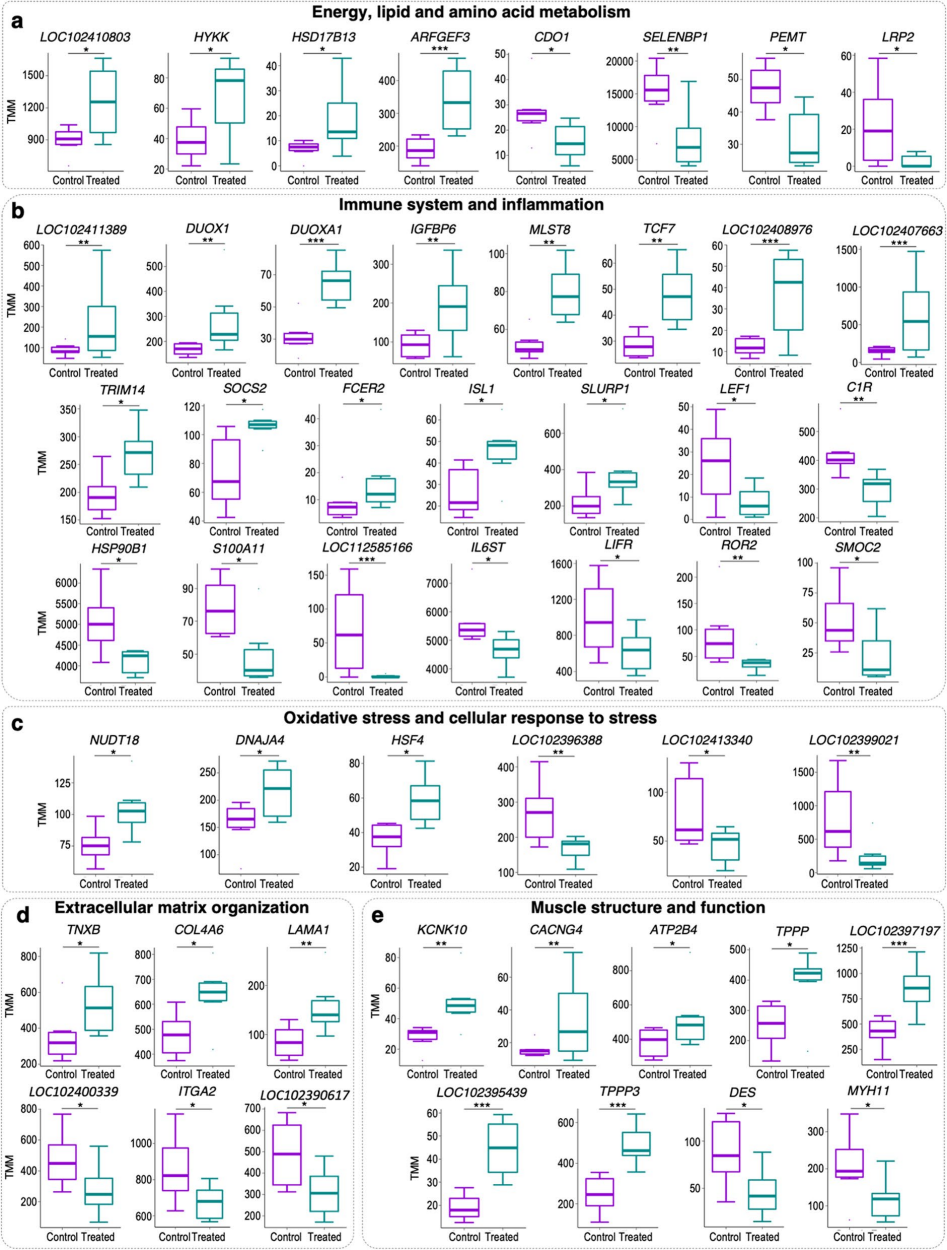
Transcriptomic data of differential expression of genes involved in selected biological processes. Boxplots illustrate the Trimmed Mean of M values (TMM) expression values of selected genes deregulated in rumen of dairy buffaloes fed a TMR + green feed (Treated) versus animals fed the TMR diet (Control) and related to energy, lipid or amino acid metabolism (**a**), immune system and inflammation (**b**), oxidative stress and cellular response to stress (**c**), extracellular matrix organization (**d**) and muscle structure and function (**e**). Whiskers show the 5–95% percentiles of the confidence intervals, the horizontal lines within each box show the medians, and outliers are shown as dots. * *p*-value < 0.05; ** *p*-value < 0.01; *** *p*-value < 0.001

Our analysis highlighted differential expression of genes encoding factors involved in energy, lipid, and amino acid metabolism (especially cysteine and methionine), in treated versus control buffaloes (Fig. 5a). We found enhanced expression of *LOC102410803* (or *Phosphoserine Aminotransferase 1, PSAT1*), and *Hydroxylysine Kinase* (*HYKK)*, and decreased levels of *cysteine dioxygenase Type 1* (*CDO1*), *SELENBP1* and *phosphatidylethanolamine N-methyltransferase* (*PEMT*) genes, encoding enzymes with a function in amino acid metabolism. Furthermore, we found up-regulation of *HSD17B13*, and *ARFGEF family member 3* (*ARFGEF3*) genes, and down-regulation of *LRP2*, encoding factors implicated in energy and lipid metabolism.

In conclusion, these findings allow to speculate that green feed has an impact on amino acid and lipid metabolism that, in turn, influence energy production.

#### Genes linked to immune system and inflammation

Green feed seems to modulate the expression of several genes encoding factors with a role in immune system or inflammation (Fig. 5b). Among them, we found enhanced expression of *dual oxidase 1 (DUOX1)*, *DUOXA1*, *IGFBP6*, *MTOR associated protein LST8 homolog* (*MLST8*), *transcription factor 7* (*TCF7*), *LOC102408976* (or *IFI27*), *LOC102407663* (or *IFI27L2*), *tripartite motif containing 14* (*TRIM14*), and *suppressor of cytokine signaling 2 (SOCS2)* genes, which are correlated with the innate or adaptative immune response. Moreover, the expression of the receptor of immunoglobulin E *FCER2*, *LOC102411389* (*VNN1*), *ISL LIM homeobox 1* (*ISL1*), and *Secreted LY6/PLAUR Domain Containing 1* (*SLURP1*) genes, which are linked to the regulation of inflammatory response, was increased. On the other side, we found reduced levels of the immune system-related *lymphoid enhancer binding factor 1* (*LEF1*) and *complement C1r* (*C1R*) genes, and inflammation-related *heat shock protein 90 beta family member 1* (*HSP90B1*), *S100 calcium binding protein A11* (*S100A11*), *LOC112585166* (or *serum amyloid A protein*-*like, SAAL1*), *interleukin 6 cytokine family signal transducer* (*IL6ST*), *leukemia inhibitory factor receptor* (*LIFR*), *ROR2*, and *SMOC2* genes.

In summary, the large number of genes with a role in immune response and inflammation, whose expression is re-modulated upon green feed administration, allows to hypothesize a beneficial effect of green diet on the animal health.

#### Genes linked to oxidative stress and cellular response to stress

Upon green feed administration, changes in the expression levels of a number of factors linked to oxidative stress and cellular response to stress have been observed (Fig. 5c). Among them, we found up-regulation of *nudix hydrolase 18* (*NUDT18*), *DnaJ heat shock protein family* (*Hsp40*) *member A4* (*DNAJA4*) and *heat shock transcription factor 4* (*HSF4*) genes, encoding factors that counteract oxidative and heat stress conditions, and down-regulation of *LOC102396388* (or *UDP glucuronosyltransferase family 1 member A9*, *UGT1A9*), *LOC102413340* (or *ATP binding cassette subfamily C member 4, MRP4/ABCC4*) and *LOC102399021* (*CYP1A1*) genes, involved in detoxifying cellular response.

Our findings prompt to hypothesize that green feed modulates the response of animals to different kind of stressful stimuli.

#### Genes linked to extracellular matrix organization (ECM)

Green feed seems to induce a significant deregulation of genes encoding factors with a function in the organization of extracellular matrix (ECM) (Fig. 5d), a complex and dynamic non-cellular scaffold that acts as a structural support for cells, and known to regulate several functions, including shape, adhesion, and migration. We found up-regulation of *collagen type IV alpha 6 chain* (*COL4A6*) and *laminin subunit alpha*-*1* (*LAMA1*) genes, and down-regulation of *LOC102400339 (*or *COL1A1),* encoding structural constituents of ECM. Moreover, in treated buffaloes, the ECM glycoprotein *tenascin XB* (*TNXB*), whose product plays a role in the expression and deposition of collagen into the ECM, was significantly up-regulated, whereas the gene encoding the *integrin subunit alpha 2 (ITGA2),* which connects the ECM with the cytoskeleton, and *LOC102390617* (or *UDP-glucose 6-dehydrogenase-like, UGDH*) gene, encoding an oxidoreductase involved in the production of extracellular matrix components, were significantly down-regulated.

Our results allow to hypothesize that green feed influences the organization of extracellular matrix in the rumen of buffaloes.

#### Genes linked to muscle structure and function

Green feed induced differential expression of several genes encoding proteins involved in muscle structure and function (Fig. 5e). We found enhanced expression of *Potassium two pore domain channel subfamily K member* (*KCNK10*), *calcium voltage*-*gated channel auxiliary subunit gamma 4* (*CACNG4*), and *ATPase plasma membrane Ca*^*2+*^*transporting 4* (*ATP2B4*), which encode factors that regulate homeostasis of Ca^2+^. Moreover, we detected up-regulation of the cytoskeleton*-*related *tubulin polymerization promoting protein* (*TPPP*), *LOC102397197* (or *TUBA1D*), *LOC102395439* (or *FHL1*), and *TPPP3* genes, and down-regulation of the *desmin* (*DES*) and *myosin heavy chain 11* (*MYH11*) genes.

Overall, changed expression of muscle-related genes suggests an impact of green feed on the muscularity of ruminal wall.

## Discussion

The present study aimed to investigate the impact of green forage on the ruminal transcriptional program through RNA-seq, which represents an excellent tool for large scale gene expression studies. Although many different bioinformatic procedures are currently available to analyze RNA-seq data, there is no consensus about which are the most reliable, and the choice of the most appropriate tool is still arduous [22]. For this reason, we approached the identification of DEGs by using two different algorithms, EdgeR and NOISeq, both considered to be robust methods [22, 23].

The EdgeR approach with a nominal *p*-values < 0.05 allowed the identification of 155 DEGs in rumen of treated versus control buffaloes. Conversely, the use of FDR-adjusted *p*-values as significance threshold did not reveal significant DEGs between treated and control groups. We hypothesized that the lack of FDR significant genes might be due to the moderate impact of green feed on gene expression, which might, in turn, be masked by the inter-individual variability of the buffaloes belonging to the same group. It is known that different feed regimens are known to induce large changes in gene expression [1]. However, the aim of our study was to understand if the solely inclusion of green forage would be responsible for differences in rumen gene expression profile, and hence we use two diets isonitrogenous and isoenergetic; therefore, we expect subtle differences between treated and control groups. In support of our hypothesis, we observed that the 155 DEGs identified with a *p*-value < 0.05 had a median absolute log2 fold change of 0.82. In addition, Barton and coworkers [24] showed that most differential expression analysis tools, including EdgeR, have lower performance at lower fold changes, as indicated by their True Positive Rate (TPR). We may hypothesize that inclusion of 30% of ryegrass green feed in the treated group was not great enough to have considerable impacts on the gene expression. Additional studies performed feeding the animals a diet with a higher proportion of green ryegrass, as well as the use of a greater number of animals, might improve the statistical power, thus emphasizing gene expression changes. However, it has been already observed that an inclusion higher than 30% of high-quality green feed in the diet of dairy cows fed TMR have reduced both the dry matter intake and milk yield [25], and hence would not be practically suitable for breeders.

The lack of FDR significant genes is suggestive of a limitation of our study, which appears statistically underpowered. However, rt-qPCR assay on 12 randomly selected DEGs identified using nominal *p*-values < 0.05, confirmed their differential gene expression observed by RNA-seq experiment and suggests a genuine impact of green feed on gene expression. On the other hand, we cannot rule out the occurrence of false positive and negative DEGs, due to the validation of only a subset of them by rt-qPCR. It is conceivable that validation of additional DEGs identified in our study should further confirm the impact of green diet on the ruminal transcriptional program.

A parallel analysis of differential expression performed using NOISeq software with a prob. ≥0.8 as significance threshold, highlighted 61 DEGs, which dropped to 11 or 1 DEGs, with prob. ≥0.9 or prob. ≥0.95, respectively. Among DEGs identified with NOISeq, only 30 genes showed a differential expression coherent with EdgeR results, 3 of which have prob. ≥0.9, and none had prob. ≥0.95. It is interesting to note that rt-qPCR assay validated the differential expression of randomly selected DEGs identified with both methods (common DEGs), while differential expression of genes highlighted exclusively with NOISeq tool, including the gene with prob. ≥0.95, was not confirmed by this assay.

In summary, in our biological context, results obtained using EdgeR software, although statistically unpowered, seems to be more reliable as compared with those obtained with NOISeq tool, and this agrees with the proposed higher performance of EdgeR in certain experimental conditions [23]. Based on these premises, we considered, for the subsequent analyses, DEGs identified with EdgeR tool, emphasizing those revealed with both approaches.

RNA-seq data, obtained using EdgeR tool, indicated that green forage impacted on the expression of several genes encoding factors implicated in the regulation of biological processes relevant for buffalo welfare, such as those related to cellular response to stress, immune system and inflammation, as well as genes encoding factors involved in rumen functionality, such as muscle structure/function, and energy, lipid, and amino acid metabolism. This evidence suggested an enhancement of rumen activity by green feed and allows to speculate a role of this diet in the production of nutraceutical molecules, whose levels might be enhanced also in milk. It was previously shown that 30% green forage increases the concentration of beneficial biomolecules, including betaine and carnitine, in milk and other dairy products in buffaloes [17, 26]. In the present study, green feed modulated the expression of genes linked to amino acid metabolism, including those related to cysteine/methionine, serine and lysine metabolism. These compounds mitigate oxidative damage in tissues by acting as precursors of anti-oxidant molecules, such as S-adenosylmethionine, hydrogen sulfide (H_2_S), taurine, and glutathione [27]. This aspect is relevant, since betaines, which have a high nutraceutical action, are the methyl donor in the cysteine/methionine pathway that converts methionine to cysteine. In particular, green feed was associated with decreased expression of *CDO1* and *SELENBP1*. Of note, decreased levels of *SELENBP1* has been detected also with NOISeq analysis, and both genes are included in the subset of DEGs randomly validated by rt-qPCR.

*CDO1* encodes the cysteine dioxygenase enzyme that has a key role in the catabolism of cysteine and taurine biosynthesis, thus regulating cysteine intracellular levels and availability [28, 29]. *SELENBP1* encodes an enzyme important for oxidation of methanethiol, a factor linked to the methionine catabolism [30]. Decreased *CDO1* expression in the rat liver was associated with high levels of many amino acids and their metabolites, including betaine [28]. As noted above, green feed increases levels of betaines in buffalo milk [17]. Based on the above findings, we speculate that green feed impacts betaine levels through the regulation of genes implicated in the cysteine/methionine pathway, such as *CDO1* and *SELENBP1*. Moreover, *PEMT,* whose expression is modulated by green feed, transcribes for the phosphatidylethanolamine N-methyltransferase, a rate-limiting enzyme of the biosynthetic pathway of choline, which is the precursor of betaine (trimethyl-glycine). Choline, in turn, participates in homocysteine production [31]. CDO1 over-expression is known to decrease cellular glutathione (GSH) levels by approximately 40% [32], thus *CDO1* down-regulation observed upon green feeding could be responsible for enhanced antioxidant activity. Since SELENBP1 is considered an “H_2_S-producing enzyme”, its reduced levels might be responsible for lower production of H_2_S which is involved in the mitochondrial transport of carnitine/acyl-carnitine [33, 34]. Green feed induced also up-regulation of *LOC102410803* (or *PSAT1)* and *HYKK. PSAT1* encodes a phosphoserine aminotransferase involved in the biosynthesis of serine, a non-essential amino acid implicated in many biological processes and crucial for the formation of glycine, cysteine, and taurine amino acids [35]. *HYKK* encodes hydroxylysine kinase that is involved in the catabolism of lysine [36]. This is related to the metabolism of carnitine since trimethyl-lysine is the main precursor of carnitine [37, 38]. We might speculate that green feed influences carnitine levels [17] by interfering with lysine metabolism.

Among genes implicated in the lipid metabolism there was up-regulation of *HSD17B13*, which encodes the 17-beta-hydroxysteroid dehydrogenase 13 enzyme that is thought to be involved in fatty acid metabolism [39], although this remains controversial. The over-expression of *HSD17B13* was associated with increased lipogenesis and triglyceride levels in the liver of mice [39]. Under-expression of *HSD17B13* in mice leads to increased levels of C16 and C18:1 acyl-carnitine, which suggest a key role of HSD17B13 in β-oxidation of fatty acids through the modulation of their mitochondrial transport [40]. It is possible that the increased expression of *HSD17B13* in buffaloes that received green forage stimulated the β-oxidation of fatty acids, influencing the transport of acyl-carnitine into the mitochondria. Moreover, it is interesting to note that HSD17B13 has also an anti-inflammatory role, since its deficiency triggers hepatic steatosis and inflammation in mice [40].

Green feed seems to impact also on energy metabolism. We found, indeed, with both EdgeR and NOISeq methods of analysis, up-regulation of *ARFGEF3,* encoding the Brefeldin A-inhibited guanine nucleotide-exchange protein 3 (BIG3), a factor that participates to the regulation of systemic glucose homeostasis, through the regulation of insulin and glucagon secretion [41, 42].

Metabolism is well known to fuel cell activities. In ruminants, for instance, amino acid metabolism is of utmost importance, since it generates several effects on animal immunity and production [43]. Our results suggest that green feed exerts a positive impact on immune system, through the modulation of the expression of genes encoding factors implicated in the immune response and inflammation. There was up-regulation of *LOC102408976* (or *IFI27*), *LOC102407663* (or *IFI27L2), TRIM14*, *DUOXA1, DUOX1, IGFBP6*, and *SLURP1* genes. *IFI27* and *IFI27L2* genes encode two factors belonging to the family of interferon-induced proteins, which are involved in both innate immune response [44] and energy metabolism [45]. *TRIM14* encodes a protein belonging to TRIM family, which are also known to promote host defense against virus infections [46]. DUOXA1 is implicated in the maturation/activation of DUOX1 that is, in turn, a NADPH oxidase known to catalyze ROS production and implicated in innate immune defense and antimicrobial function [47]. IGFBP6 is involved in the immune response, with both pro- and anti-inflammatory roles [48], and its expression has been reported to be enhanced following an increase in the fiber/starch ratio in cattle diet [49]. SLURP1 is a nicotinergic peptide known to exert an anti-inflammatory effect in the human intestinal epithelial cells [48]. Notably, up-regulation of *DUOXA1, DUOX1, IGFBP6*, and *SLURP1* has been highlighted also with NOISeq analysis. Furthermore, we found up-regulation of *SIM2* that encodes a factor involved in the anti-microbial response in the gastrointestinal tract by regulating the expression of anti-microbial peptides and defensins [50]. Overall, these data seem to suggest a beneficial role of green feed, by stimulating the immune system and protecting against pathogens.

It is known that the rumen functionality depends on the microbiota composition. The cross-talk between rumen microbiota and innate immune cells in the ruminal epithelium may contribute to the balance of immune tolerance and inflammatory response in the rumen, which is partly dependent on metabolites and, thus, diet [51, 52]. High amount of vaccenic acid, the dietary CLA precursor, were observed in milk of buffaloes receiving green feed [17]. This is of particular interest considering that CLA exerts an anti-inflammatory function in sheep ruminal epithelial cells through the inhibition of pro-inflammatory cytokines production and the regulation of cell proliferation and lipid metabolism, which are potentially related to injury repair and immune response [53]. Accordingly, we found that green feed modulated the expression of many genes with a role in the inflammation. Among the down-regulated genes, we found *LOC112585166* (or *SAAL1*), *ROR2*, *SMOC2,* and *S100A11*, which are associated with pro-inflammatory activity [54–57]. Of note, down-regulation of *serum amyloid A1* (*SAA1)* was observed in rumen papillae of dairy cow fed green forage [58], which underlines the susceptibility of its expression to the diet. On the other hand, *ISL1* gene, whose expression was increased upon green feed administration, is known to negatively regulates the inflammatory response [59]. Additionally, we found up-regulation of *LOC102411389* (or *VNN1*), encoding the enzyme pantethinase, which is involved in the production of pantothenic acid (vitamin B5) and coenzyme A (CoA). *VNN1* has been linked to inflammation, stress response, lipid metabolism and energy production [60, 61]. Of note, *VNN1* gene has been previously reported to be differentially expressed in ruminal epithelium of heifers fed low or high grain diet [62]. Among the above discussed inflammation-related genes, *ROR2*, *SMOC2,* and *VNN1* were differentially expressed also with NOISeq analysis. Altogether, these data allow to hypothesize an anti-inflammatory role of green forage, through the modulation of genes encoding pro- and anti-inflammatory factors.

Cells are continuously exposed to several stressful conditions that could negatively impact on cell metabolism, promote cell and tissue damages and contribute to the onset of several pathologies. Among the main sources of stress there are the oxidative stress, a detrimental condition arising from imbalanced levels of reactive oxygen species (ROS) and antioxidants, and stress conditions caused by excessive heat, cold or UV light. Green feed induced changes in the expression of genes linked to oxidative stress and cellular response to stress. Particularly notable was up-regulation of genes whose products counteract stressful conditions, such as *NUDT18*, *DNAJA4* and *HSF4* genes. *NUDT18* catalyzes the hydrolysis of 8-oxo-dGTP - one of the best characterized DNA damages caused by oxidative stress - to produce the monophosphate form 8-oxo-dGMP (odGMP), which is unusable for DNA synthesis, thus preserving cells from oxidation-mediated mutations [63]; DNAJA4 belongs to the family of heat shock proteins HSP40, which cooperate with HSP70 chaperones to counteract protein misfolding and aggregation in stress conditions [64]; *HSF4* gene encodes a factor that modulates the expression of heat shock proteins under stressful conditions [65] and exerts a protective effect against oxidative stress [66]. These findings suggest that green feed may exert an anti-oxidant function, by positively modulating factors that counteract stressful stimuli. In addition, we found down-regulation of genes encoding factors involved in detoxifying cellular response, such as *LOC102396388* (or *UGT1A9*), coherently deregulated with both EdgeR and NOISeq approaches, *LOC102413340* (or *MRP4/ABCC4*), and *LOC102399021* (or *CYP1A1*), which might arise from the healthy nature of the green feed. Noteworthy, the expression of *HSF4*, *LOC102396388*, *LOC102413340*, and *LOC102399021* has been reported to be modulated by different diet composition [62, 67–69].

Extracellular matrix has a crucial role in proliferation, differentiation, adhesion and inflammation. Many reports underlined a diet-related re-modulation of extracellular matrix [70–72], according with our data, which suggest an impact of green forage on the expression of ECM-related genes. Among them, we found up-regulation of *LAMA1* and *COL4A6* and down-regulation of *LOC102400339* (or *COL1A1*) and *LOC102390617* (or *UGDH*). Of note, *LAMA1*, *LOC102400339*, and *LOC102390617* showed similar deregulation with NOISeq analysis. COL4A6, COL1A1 and LAMA1 are three of the major structural constituents of ECM [73, 74], whereas UGDH is an enzyme involved in the production of glycosaminoglycans and proteoglycans of ECM [75]. These findings allow to speculate that green forage influence the ECM modelling and thus, the establishment of the structure and the function of the cells.

The ruminal wall has an important role in nutrient uptake and represents a barrier against pathogens and mechanical injuries [76]. Buffaloes that received green feed showed changes in the expression of many genes encoding factors associated with muscle structure and function. Of particular interest is the increased expression of *KCNK10, CACNG4,* and *ATP2B4* genes, encoding factors that modulate the homeostasis of Ca^2+^ [77–80], a second messenger important for muscle contraction and differentiation, which allows to suppose that green feed might stimulate the ruminal muscular function. Furthermore, green feed modulated, with a different direction, genes encoding structural components of the cytoskeleton, such as *MYH11, DES,* and *LOC102397197 (*or *TUBA1D)*, and factors promoting its assembling, such as *TPPP*, *TPPP3*, thus suggesting an impact of this diet on muscular structure. *LOC102397197* and *TPPP3* genes resulted differentially expressed also using NOISeq tool. Interestingly, *DES* expression was previously shown to be modulated by diet [81].

## Conclusions

Although the low statistical power of our study suggests a limitation of this work and point out the need of additional investigations, we provide, for the first time, a broad overview of the different biological processes that seems to be influenced by a green feed diet in buffalo rumen epithelium. Taking advantage of a transcriptomic approach, we deepened our understanding on the response of ruminal metabolism, activity, and physiology to different diet regimens. This work adds a piece of knowledge on the potential effects of the diet on molecular processes linked to the animal welfare and production of nutraceutical molecules. This study provides clues for future research on ruminants, and novel aspects that should be considered in addition to rumen fermentation, such as immune activity and oxidative response, for the setup of feeding strategy focused to enhance animal welfare and production. Our findings will pave the way for the optimization of novel strategies based on the precision feeding system, respectful of the ethologic and physiologic needing of the animals. However, additional studies are needed to further confirm the impact of green forage on ruminal transcriptional program, possibly through the validation of additional DEGs here identified and the increase of sample size. Moreover, further omics investigations, including methylomic studies, might allow to decipher the molecular impact of green feed at multiple layers.

## Materials and methods

### Animals, dietary treatment, and ruminal tissue collection

All experimental procedures were performed according to the European Directive 2010/63/EU and the Italian Legislative Decree No. 26 dated 4th March 2014 and received institutional approval from the Ethical Animal Care and Use Committee of the University of Naples “Federico II” (Protocol No. 25532–2022). The study was carried out over 60 days at a commercial buffalo dairy in southern Italy, by using Italian Mediterranean dairy buffaloes (n 12; 8.2 ± 0.6 years old), for which the slaughter was already scheduled. The animals were maintained in pens with a concrete floor and were milked twice daily in the morning and afternoon and had undergone a 14-day adaptation period before beginning the trial. Buffaloes were randomly assigned to two equal groups (Control and Treated) according to parity (5.2 ± 0.4 vs 5.3 ± 0.7 in treated vs control group, respectively; *P* > 0.05, two-tailed Student’s t-test), and average milk production (6.0 ± 0.7 vs 6.8 ± 0.7 kg/day in treated vs control group, respectively; P > 0.05, two-tailed Student’s t-test), whose values were recorded in the 10 days before commencement of the study. The diet of control buffaloes was a total mixed ration (TMR) whilst treated buffaloes received TMR + green feed which comprised ryegrass (approximately 30% of the diet, Table 5).

The forage was just ryegrass at the re-blossoming stage cut twice a day to avoid any fermentation and immediately put in the mixing wagon, with no storage and administered to animals. The forage to concentrates ratio of control buffaloes was 56:44 and that of treated buffaloes was 69:31. The two diets were isonitrogenous and isoenergetic and differed only in the inclusion of green feed in treated buffaloes (Table 5). Animals were fed twice daily: in the morning and in the evening. Refusals were recorded and then removed. Animals were euthanized all together by penetrating captive bolt, according to the AVMA Guidelines for the Humane Slaughter of Animals, following procedures approved by the Ethical Animal Care and Use Committee of the University of Naples “Federico II”. Ruminal wall biopsies were collected, then, they were immediately washed with cold PBS, and stored at − 80 °C before RNA extraction.

**Table 5:** Feed and chemical composition of the buffalo diets without (Control) or with (Treated) 30% green ryegrass

Item	Control buffaloes	Treated buffaloes
*Component*	*Amount (kg of feed)*	
Ryegrass	–	25
Corn silage	18	13
Alfalfa hay	5	1
Soybean meal (48%)	1.6	–
Concentrate	4.4	4
Corn meal	1	1.8
Hydrogenated fats	0.3	0.3
Calcium Carbonate	0.1	–
Salt 1:3	0.1	0.1
Vitamins	0.1	–
*Composition (% dry matter intake)*		
Dry matter	16.5	16.6
Crude protein	14.7	14.7
Fat	6.0	7.0
NDF	36.8	36.8
ADF	21.2	19.5
NSC	33.8	34.7
Starch	18.8	18.8
Ash	8.8	6.8
Calcium	0.9	1
Phosphorus	0.4	0.4
MFU	0.93	0.93

*NDF* Neutral detergent fiber, *ADF* Acid detergent fiber, *NSC* Non-structural carbohydrates, *MFU* Milk forage units

### Isolation of total RNA from ruminal wall

Total RNA was isolated from 100 to 200 mg of frozen ruminal wall tissue of 12 dairy buffaloes, of which 6 were fed a TMR (control group) and 6 received TMR + green feed (treated group). RNA was extracted using the EuroGold TriFast (EuroClone, Pero, Milano, Italy) reagent, according to the manufacturer’s instructions. Tissue samples were homogenized in 1 ml of TriFast reagent by using TissueLyser (Qiagen, Hilden, Germany), then, RNA samples were treated with Turbo DNA-freeTM kit (Ambion, Austin, TX, USA) to remove genomic DNA contaminant, according to the manufacturer’s instructions. The quality and the quantity of RNA samples were determined using Agilent 2100 Bioanalyzer (Agilent Technologies, Santa Clara, CA). Samples with an RNA integrity number (RIN) greater than 7.0 were used for library construction. RNA samples were stored at − 80 °C until further processing.

### RNA-seq library preparation and sequencing

RNA-seq library preparation and sequencing were carried out at Macrogen (Seoul, Korea). Briefly, the RNA-seq libraries were prepared using TruSeq Stranded Total RNA Sample Prep kit (Illumina, San Diego, CA) according to the manufacturer’s recommendations, using 1 μg of total RNA as input. Starting RNA samples and final RNA libraries were quantified by using the Qubit 2.0 Fluorometer (Invitrogen, Carlsbad, CA) and quality tested by Agilent 2100 Bioanalyzer RNA Nano assay (Agilent technologies, Santa Clara, CA). Libraries were then processed with Illumina cBot for cluster generation on the flow cell, following the manufacturer’s instructions and sequenced (paired end sequencing, 150 bp, 30 M reads per sample) at the multiplexing level requested on NovaSeq6000 (Illumina, San Diego, CA). The CASAVA 1.8.2 version of the Illumina pipeline was used to process raw data for both format conversion and de-multiplexing.

### RNA-seq reads processing and mapping

Bioinformatic analysis were carried out by Sequentia Biotech (Barcelona, Spain). The quality of raw RNA-seq reads was assessed with the software FastQC v0.11.5 (https://www.bioinformatics.babraham.ac.uk/projects/fastqc/). Adapters and low-quality bases were then removed using Trimmomatic v0.39 setting a minimum base quality of 25, minimum length of 35 bp and leaving the other options as default [82]. Trimmed reads were then aligned to the buffalo genome (*Bubalus bubalis*, version UOA_WB_1 from NCBI) with STAR aligner v2.5.2b [83] using default parameters. Read summarization to produce gene counts matrix was performed with featureCounts v2.0.0 [84] with the following parameters: -Q 30 -s 2 -p.

### Identification of differentially expressed genes

Differential expression analysis was carried out using two approaches. With the first one, lowly expressed genes and those showing high variability were filtered out using the HTSFilter R package [85] using the TMM normalization [86], which selected a threshold of 41.64 TMM. Sample clustering was assessed performing a Principal Component Analysis (PCA) on the TMM normalized values after the HTSFilter step. Differentially expressed genes were detected using EdgeR [87] by nominal *p*-value < 0.05. With the second approach, lowly expressed genes were filtered out using NOISeq [88] setting a threshold of 1 CPM, then the ARSyN correction was applied on the log2 TMM normalized values using the same package, in order to reduce experimental noise. Finally, the NOISeq function was used to perform the statistical function assigning a probability of differential expression to each gene. Genes showing a probability value higher than or equal to 0.8, 0.9 and 0.95 were considered for further analyses.

### Functional analysis

The differentially expressed genes were split into up- and down-regulated genes and then a Gene Ontology Enrichment Analysis was performed on each list using an in-house developed script based on the methods used by the AgriGOv2 algorithm [89]. Significantly Enriched Gene Ontology terms were identified applying an FDR (*p*-value adjusted by Benjamini-Hochberg correction) filter of 0.05 [90].

### Reverse-transcription quantitative PCR (RT-qPCR) validation of selected DEGs

To validate RNA-seq results, DEGs were randomly selected and analyzed by reverse-transcription qPCR (rt-qPCR). cDNA was obtained by reverse transcription of 500 ng of total RNA using the SuperScript II First-Strand Synthesis system (Life Technologies, Carlsbad, CA, USA), following the manufacturer’s instructions. The obtained cDNA was, then, amplified by qPCR with SsoAdvance Universal SYBR Green supermix (Bio-Rad, Hercules, CA, USA) on a CFX Opus Real-Time PCR system (Bio-Rad), according to the manufacturer’s protocols. The 2^-ΔΔCq^ method was used to determine the relative quantitative levels, and the data were normalized with respect to the expression of β-actin, which showed a similar distribution of FPKM values with no statistically significant difference between the two experimental groups (Wilcoxon Test, *p* = 0.96; data not shown). List of primer sequences used in this study is reported in the Additional file 5: Table S5.

## Supplementary Information

**Additional file 1: Table S1.** Summary of the mapping information for each sample. CTRL: Control buffaloes; TREAT: Treated buffaloes.

**Additional file 2: Table S2.** List of genes expressed in rumen of treated and control dairy buffaloes.

**Additional file 3: Table S3.** List of DEGs identified with EdgeR and NOISeq analysis in treated buffaloes in comparison with control animals.

**Additional file 4: Table S4.** List of GO terms significantly enriched in genes up-regulated and down-regulated in treated buffaloes in comparison with control animals.

**Additional file 5: Table S5.** List of primers used in rt-qPCR experiments.

## Data Availability

The RNA-sequencing dataset supporting the conclusions of this article is available in the GEO repository (accession number GSE207815).

## Abbreviations

*ABCC4*: Multidrug resistance-associated protein 4-like
*ACSS3*: Acyl-CoA synthetase short-chain family member 3
*ARFGEF3*: Brefeldin A-inhibited guanine nucleotide-exchange protein 3 Family Member 3
*ATP2B4*: ATPase plasma membrane Ca^2+^ transporting 4
BIG3: Brefeldin A-inhibited guanine nucleotide-exchange protein 3
*BMP6*: Bone morphogenetic protein 6
*C1R*: Complement component 1 subcomponent R
*CACNG4*: Calcium voltage-gated channel auxiliary subunit gamma 4
*CDO1*: Cysteine Dioxygenase Type 1
CLA: Conjugated linoleic acid
*COL1A1*: Collagen alpha-1(I) chain
*COL4A6*: Collagen type IV alpha 6 chain
*Cox7C*: Cytochrome C oxidase subunit 7C
*CYP1A1*: Cytochrome P450 1A1
DEGs: Differentially expressed genes
*DES*: Desmin
*DNAJA4*: DnaJ heat shock protein family member A4
*DUOX1*: Dual oxidase 1
*DUOXA1*: Dual oxidase maturation factor 1 isoform X2
ECM: Extracellular matrix
*FCER2*: Low affinity immunoglobulin epsilon Fc receptor
*FHL1*: Four and a half LIM domains 1
*FRAS1*: Fraser extracellular matrix complex subunit 1
GO: Gene ontology
GOEA: Gene ontology enrichment analysis
H_2_S: Hydrogen sulfide
*GSTA1*: Glutathione S-transferase A1
*HSD17B13*: 17-beta-hydroxysteroid dehydrogenase 13
*HSF4*: Heat Shock Transcription Factor 4
Hsp40: Heat Shock Protein 40
*HSP90B1*: Heat Shock Protein 90 Beta Family Member 1
*HYKK*: Hydroxylysine Kinase
*IFI27*: Interferon Alpha Inducible Protein 27
IFI27L2: Interferon alpha-inducible protein 27-like protein 2
*IFI44L*: Interferon-induced protein 44-like
*IGFBP6*: Insulin-like growth factor-binding protein 6
*IL6ST*: Interleukin 6 cytokine family signal transducer
*ISL1*: ISL LIM homeobox 1
*ITGA2*: Integrin subunit alpha 2
*ITGA8*: Integrin alpha-8
*KCNK10*: Potassium two pore domain channel subfamily K member
*LAMA1*: Laminin Subunit Alpha-1
*LEF1*: Lymphoid Enhancer Binding Factor 1
*LIFR*: *Leukemia inhibitory factor receptor*
*LRP2*: Low-density lipoprotein receptor-related protein 2
*MLST8*: MTOR associated protein, LST8 homolog
*MRP4/ABCC4*: ATP Binding Cassette Subfamily C Member 4
*MUC15*: Mucin-15
*MYH11*: Myosin heavy chain 11
*NDEL1*: Nuclear distribution protein nudE homolog 1-like
*NUDT18*: Nudix hydrolase 18
odGMP: 8-oxo-dGMP
*OR51E1*: Olfactory receptor 51E1
*PEMT*: Phosphatidylethanolamine N-Methyltransferase
*PNLDC1*: Poly(A)-specific ribonuclease PNLDC1
*PSAT1*: Phosphoserine aminotransferase 1
RNA-seq: RNA sequencing
*ROR2*: Tyrosine-protein kinase transmembrane receptor
ROS: Reactive oxygen species
rt-qPCR: Reverse-transcription quantitative PCR
*S100A11*: S100 calcium binding protein A11
*SAA1*: Serum Amyloid A1
*SAAL1*: *Serum amyloid A protein-like*
SAM: S-adenosyl methionine
SCFAs: Short-chain fatty acids
*SELENBP1*: Selenium Binding Protein 1
*SIM2*: Single-minded homolog 2 protein
*SLURP1*: *Secreted LY6/PLAUR Domain Containing 1*
*SMOC2*: SPARC-related modular calcium-binding protein 2
*SOCS2*: Suppressor of cytokine signaling 2
*SYNPO2*: Synaptopodin-2
*TCF7*: Transcription factor 7
*TCN1*: *Transcobalamin-1*
*TENM3*: Teneurin-3
*TSKS*: Testis-specific serine kinase substrate
TMM: Trimmed Mean of M values
TMR: Total mixed ration
*TNXB*: Tenascin XB
*TPPP*: Tubulin polymerization promoting protein
*TPPP3*: Tubulin polymerization promoting protein family member 3
TPR: True Positive Rate
*TRIM14*: Tripartite motif containing 14
*TTPA*: Alpha tocopherol transfer protein
*TUBA1D*: Tubulin Alpha 1d
*UGT1A9*: UDP Glucuronosyltransferase Family 1 Member A9
*VNN1*: Vanin 1
*WFDC18*: WAP four-disulfide core domain protein 18-like
*WTIP*: Wilms tumor protein 1-interacting protein
